# Antifungal Prophylaxis in Acute Myeloid Leukemia: New Drugs, New Challenges? Summary of the EHA Guideline on Antifungal Prophylaxis in Adult Patients With Acute Myeloid Leukemia Treated With Novel-targeted Therapies

**DOI:** 10.1097/HS9.0000000000000742

**Published:** 2022-06-17

**Authors:** Jannik Stemler, Oliver A. Cornely

**Affiliations:** 1 Faculty of Medicine and University Hospital Cologne, Translational Research, Cologne Excellence Cluster on Cellular Stress Responses in Aging-Associated Diseases (CECAD), University of Cologne, Germany; 2 Department I of Internal Medicine, Center for Integrated Oncology Aachen Bonn Cologne Duesseldorf (CIO ABCD) and Excellence Center for Medical Mycology (ECMM), Faculty of Medicine and University Hospital Cologne, University of Cologne, Germany; 3 German Centre for Infection Research (DZIF), Partner Site Bonn-Cologne, Germany; 4 Faculty of Medicine and University Hospital Cologne, Clinical Trials Centre Cologne (ZKS Köln), University of Cologne, Germany

Patients with acute myeloid leukemia (AML) belong to the highest risk group for developing invasive fungal disease (IFD) at diagnosis or during treatment course. Relevant entities of IFD in this population include invasive candidiasis and candidemia mostly acquired through fungal translocation of commensal yeasts from the lumen of the gastrointestinal tract via the mucosal barrier, whereas invasive mold infections are acquired through airborne inhalation of fungal conidia, airway germination, and subsequent dissemination. These entities affect profoundly neutropenic patients. Therefore, triazole-based antifungal prophylaxis for mold coverage has been a cornerstone for clinical management in this patient population for decades, with results from randomized controlled trials (RCT) supporting its use in prevention of IFD and reducing mortality with low numbers-needed-to-treat.^1,2^

Although novel-targeted therapies have recently become available with promising data to improve the outcome of AML, their metabolism pathways interact with antifungals of the azole class. This poses significant challenges in patient management.^3^ Clinical questions at this crossroad comprise (i) if antifungal prophylaxis is to be implemented depending on novel agent and status of disease, (ii) the selection of antifungal agent and duration of its use, and (iii) the role of potential drug-drug interactions (DDI) for toxicity.^4,5^ The latter has been an upcoming issue in recent years as many novel available targeted therapies, like venetoclax, FLT3-inhibitors (midostaurin, gilteritinib, quizartinib, etc.), or IDH-inhibitors (ivosidenib, enasidenib) are substrates of the cytochrome p450 enzyme system to a varying extent, whereas triazole antifungals like posaconazole and voriconazole are potent inhibitors of this system.^6^

To address those questions, EHA gathered an expert group in 2019 to give recommendations for clinical management in cooperation with the Cochrane Haematology Group. For the resulting guideline, studies including all relevant novel-targeted agents and antifungal agents were reviewed, summarized and—depending on available studies—evidence-based or consensus-based recommendations were phrased.

Despite the obvious lack of high-quality studies assessing the use of antifungal prophylaxis in this specific setting, evidence-based recommendations were phrased for the hypomethylating agents (HMA) azacitidine and decitabine, as well as for venetoclax, gemtuzumab-ozogamicin, and midostaurin. Consensus-based statements were given for dasatinib, gilteritinib, glasdegib, idasanutlin, ivosidenib, lestaurtinib, and sorafenib, generally with low to very low certainty of evidence.^7^ For some AML drugs, no recommendation was possible due to scarce data and other agents, such as etrenapopt, olutasidenib, and others, were not considered since even the clinical trial data for safety and efficacy had not been available when this guideline was endeavored.

Generally, the use of antifungal prophylaxis is indicated in fit patients with AML during remission-induction chemotherapy (RIC), as recommended by former guidelines, also if combined with novel agents, for example, FLT3-inhibitors, especially in the context of long-lasting neutropenia.^8-10^ Since incidence of IFD in patients with AML not treated with intensive RIC has long been suggested to be lower, treating physicians do not consider this a scenario to administer antifungal prophylaxis. However, retrospective studies have shown an IFD incidence of up to 8%, being high enough to consider administering prophylaxis to prevent disease and premature death from IFD.^11^ In monotherapy settings, for example, with gilteritinib in relapsed/refractory AML, where duration of neutropenia cannot be estimated or is due to the underlying disease status, antifungal prophylaxis is also recommended. The only situation with robust underlying data from clinical studies to support dose reduction is the treatment with a combination of HMA and venetoclax with a recommendation to administer antifungal prophylaxis with a triazole and reduce the venetoclax dose by at least 75% with real-life data showing similar durations of neutropenia and thrombocytopenia as compared to administration of venetoclax without an azole.^12^ However, several retrospective studies show that potential DDI can play a role and their impact is not yet fully defined, such as in the case of midostaurin and gilteritinib. Further clinical concepts and their investigation under real-life conditions need to determine their use to support dose adaptations of the AML agent and the administration or omission of antifungal prophylaxis.^13–15^ Administration of other prophylactic antifungal agents, such as echinocandins, is more and more often considered in case of potential DDI. However, for these agents evidence is sparse and they are not lincensed for this indication in patients with AML and through their daily IV administration are a less feasible option for the outpatient setting in which many patients with orally administered targeted AML therapies are treated. Therefore, the strongest recommendation for antifungal prophylaxis remains for a mold-active triazole-based approach.^1,7,9^

If the recommendation was “either for or against antifungal prophylaxis,” this should guide the treating physician to taking the decision context-dependent, that is, according to the patient’s individual scenario, such as expected duration of neutropenia, history of IFD or local epidemiology of IFD.

The published recommendations also have limitations that remain a future challenge in the context of diversifying treatment settings for AML, emerging fungal pathogens and antifungal resistance, and different management approaches to prevent IFD. The clinical setting of a combination of any novel AML agent and their impact on development of IFD was not considered. Furthermore, the guideline’s scope was narrowed to mainly evaluate antifungal medication for prophylaxis and to respond concerns regarding potential DDI emphasizing the pharmacological perspective. Other approaches to reduce the risk of IFD, including general infection control measures like hand hygiene compliance, bundle-based care protocols for central venous access devices or reduction of environmental fungal conidia exposure, were not discussed. As a more general remark, antifungal stewardship principles should be implemented in any patient receiving an antifungal to determine the appropriate duration of exposure.

The EHA guidance also includes a research agenda. Further investigations in the field may comprise RCTs to evaluate efficacy of antifungal prophylaxis in patients with AML not treated with intensive RIC, as well as the assessment of real-life utilization of antifungal prophylaxis in patients with AML. The incidence of IFD in this high-risk population remains not well-defined and should be assessed in registries. Drug metabolization and thereby cytochrome p450-associated potential DDI including polymorphisms in the individual patient may gain importance for determination of the optimal cancer management. In this context, therapeutic drug monitoring (TDM) may play an increasing role to assess clinically relevant DDI by monitoring drug levels of both the antifungal, where TDM in a therapeutic setting is already a standard-of-care, and the antileukemic drugs. Further, this may even enable clinicians to adapt and personalize the dose according to measured level to finally optimize therapeutic effect and reduce toxicity of both drugs and thereby harnessing the maximum benefit for the individual patient.

**Table 1.** Summary of Recommendations Regarding Antifungal Prophylaxis for Clinically Relevant Antileukemic Drugs

Drug	Clinical Setting	AFP Recommendation	Evidence-Based or Consensus-Based Recommendation	Comment
**5-Azacitidine**	Induction and maintenance, generally combined with bcl-2 inhibitors	Conditional for AFP	Evidence-based with low certainty of evidence	AFP should be considered in heavily pretreated patients, as secondary prophylaxis in patients with previous IFI and patients with long-lasting neutropenia.
**Decitabine**	Induction and maintenance, generally combined with bcl-2 inhibitors	Conditional for AFP	Consensus-based	Data regarding incidence and potential benefit of AFP was also extrapolated from patients treated with azacitidine.
**Enasidenib**	Monotherapy or combination therapy with RIC or other agents	No recommendation	Consensus-based	No recommendation was given due to limited clinical use and documentation of IFI. Close monitoring for potential DDI and QTc interval.
**Gemtuzumab-Ozogamicin**	During RIC in combination with intensive chemotherapy	Conditional for AFP	Evidence-based with low certainty of evidence	Strong recommendation for AFP during RIC.
**Gilteritinib**	Monotherapy in relapsed or refractoy AML	Either for/against AFP	Consensus-based	In monotherapy setting, no benefit of AFP was documented. Triazole AFP should be considered in heavily pretreated patients.
**Glasdegib**	Monotherapy or in combination with LDAC	Conditional against AFP	Consensus-based	Close monitoring for potential DDI and QTc interval.
**Ivosidenib**	Monotherapy or combination therapy with RIC or other agents	Either for/against AFP	Consensus-based	Conditional recommendation to reduce the dose to 250mg/day if concomitant use to strong CYP43A4 inhibitors. Close monitoring for potential DDI and QTc interval.
**Midostaurin**	During RIC in combination with intensive chemotherapy or as maintenance monotherapy	Conditional for AFP	Evidence-based with low certainty of evidence	Strong recommendation for AFP during RIC. Individual decision for or against antifungal prophylaxis when administered as monotherapy. Close monitoring for potential DDI and QTc interval.
**Sorafenib**	Maintenance monotherapy or during RIC in combination with intensive chemotherapy	Conditional for AFP	Consensus-based	Strong recommendation for triazoles during RIC treatment.
**Venetoclax**	Induction and maintenance in combination with hypomethylating agents or other drugs	Conditional for AFP	Evidence-based with low certainty of evidence	Prefer AFP with triazoles during induction treatment. Reduce venetoclax dose to 70mg when using posaconazole or voriconazole concomitantly.
**Quizartinib**	During RIC in combination with intensive chemotherapy	Conditional for AFP	Consensus-based	Strong recommendation for AFP during RIC with a dose reduction of quizartinib if triazole prophylaxis is used. In monotherapy setting, recommendation against AFP.

AML = acute myeloid leukemia; AFP = antifungal prophylaxis; DDI = drug-drug interactions; HMA = hypomthylating agents; IFI = invasive fungal infection; LDAC = low-dose cytarabine;RIC = remission-induction chemotherapy.

## DISCLOSURES

JS has received research grants by the Ministry of Education and Research (BMBF) and Basilea Pharmaceuticals Inc.; has received speaker honoraria by Pfizer Inc.; and has received travel grants by German Society for Infectious Diseases (DGI e.V.) and Meta-Alexander Foundation. OAC reports grants or contracts from Amplyx, Basilea, BMBF, Cidara, DZIF, EU-DG RTD (101037867), F2G, Gilead, Matinas, MedPace, MSD, Mundipharma, Octapharma, Pfizer, Scynexis; Consulting fees from Abbvie, Amplyx, Biocon, Biosys, Cidara, Da Volterra, Gilead, Matinas, MedPace, Menarini, Molecular Partners, MSG-ERC, Noxxon, Octapharma, Pardes, PSI, Scynexis, Seres; Honoraria for lectures from Abbott, Al-Jazeera Pharmaceuticals, Astellas, Grupo Biotoscana/United Medical/Knight, Hikma, MedScape, MedUpdate, Merck/MSD, Mylan, Pfizer; Payment for expert testimony from Cidara; Participation on a Data Safety Monitoring Board or Advisory Board from Actelion, Allecra, Cidara, Entasis, IQVIA, Janssen, MedPace, Paratek, PSI, Pulmocide, Shionogi; A patent at the German Patent and Trade Mark Office (DE 10 2021 113 007.7); Other interests from DGHO, DGI, ECMM, ISHAM, MSG-ERC, Wiley.

## References

[1] Cornely OA Maertens J Winston DJ. Posaconazole vs. fluconazole or itraconazole prophylaxis in patients with neutropenia. N Engl J Med. 2007;356:348–359. 17251531 10.1056/NEJMoa061094

[2] Cornely OA Ullmann AJ. Numbers needed to treat with posaconazole prophylaxis to prevent invasive fungal infection and death. Clin Infect Dis. 2008;46:1626–1627; author reply 1627. 18419501 10.1086/587177

[3] Kantarjian H Short NJ DiNardo C. Harnessing the benefits of available targeted therapies in acute myeloid leukaemia. Lancet Haematol. 2021;8:e922–e933. 34687602 10.1016/S2352-3026(21)00270-2 PMC8996707

[4] Coussement J Lindsay J Teh BW. Choice and duration of antifungal prophylaxis and treatment in high-risk haematology patients. Curr Opin Infect Dis. 2021;34:297–306. 34039878 10.1097/QCO.0000000000000737

[5] Lindsay J Teh BW Micklethwaite K. Azole antifungals and new targeted therapies for hematological malignancy. Curr Opin Infect Dis. 2019;32:538–545. 31688198 10.1097/QCO.0000000000000611

[6] Brüggemann RJ Verheggen R Boerrigter E. Management of drug-drug interactions of targeted therapies for haematological malignancies and triazole antifungal drugs. Lancet Haematol. 2022;9:e58–e72. 34890539 10.1016/S2352-3026(21)00232-5

[7] Stemler J de Jonge N Skoetz N. Antifungal prophylaxis in adult patients with acute myeloid leukaemia treated with novel targeted therapies: a systematic review and expert consensus recommendation from the European Hematology Association. Lancet Haematol. 2022;9:e361–e373. 35483397 10.1016/S2352-3026(22)00073-4

[8] Maertens JA Girmenia C Brüggemann RJ. European guidelines for primary antifungal prophylaxis in adult haematology patients: summary of the updated recommendations from the European Conference on Infections in Leukaemia. J Antimicrob Chemother. 2018;73:3221–3230. 30085172 10.1093/jac/dky286

[9] Mellinghoff SC Panse J Alakel N. Primary prophylaxis of invasive fungal infections in patients with haematological malignancies: 2017 update of the recommendations of the Infectious Diseases Working Party (AGIHO) of the German Society for Haematology and Medical Oncology (DGHO). Ann Hematol. 2018;97:197–207. 29218389 10.1007/s00277-017-3196-2 PMC5754425

[10] Taplitz RA Kennedy EB Bow EJ. Antimicrobial prophylaxis for adult patients with cancer-related immunosuppression: ASCO and IDSA Clinical Practice Guideline Update. J Clin Oncol. 2018;36:3043–3054. 30179565 10.1200/JCO.18.00374

[11] Aldoss I Dadwal S Zhang J. Invasive fungal infections in acute myeloid leukemia treated with venetoclax and hypomethylating agents. Blood Adv. 2019;3:4043–4049. 31816059 10.1182/bloodadvances.2019000930 PMC6963254

[12] Agarwal SK DiNardo CD Potluri J. Management of venetoclax-posaconazole interaction in acute myeloid leukemia patients: evaluation of dose adjustments. Clin Ther. 2017;39:359–367. 28161120 10.1016/j.clinthera.2017.01.003

[13] Aleissa MM Alshehri BS Gonzalez-Bocco IH. Triazole antifungal use for prophylaxis and treatment of invasive fungal diseases for patients receiving gilteritinib. Leuk Res. 2021;108:106610. 34048999 10.1016/j.leukres.2021.106610

[14] Ouatas T Duval V Sinclair K Berkowitz N. Concomitant use of midostaurin with strong CYP3A4 inhibitors: an analysis from the ratify trial. Blood. 2017;130(Suppl 1):3814.

[15] Stemler J Koehler P Maurer C. Antifungal prophylaxis and novel drugs in acute myeloid leukemia: the midostaurin and posaconazole dilemma. Ann Hematol. 2020;99:1429–1440. 32514626 10.1007/s00277-020-04107-1 PMC7316674

